# Exploring variation in surgical practice: does surgeon personality influence anastomotic decision-making?

**DOI:** 10.1093/bjs/znac200

**Published:** 2022-07-19

**Authors:** Carly N Bisset, Eamonn Ferguson, Ewan MacDermid, Sharon L Stein, Nuha Yassin, Nicola Dames, Deborah S Keller, Raymond Oliphant, Simon H Parson, Jennifer Cleland, Susan J Moug

**Affiliations:** Department of General Surgery, Royal Alexandra Hospital, Paisley, UK; Department of Medical Education, University of Aberdeen, Aberdeen, UK; Department of Psychology, University of Nottingham, Nottingham, UK; Faculty of Medicine and Health, University of Sydney, Sydney, Australia; Department of Colorectal Surgery, Bankstown-Lidcombe Hospital, Australia University of Sydney, Sydney, NSW, Australia; UHRISES: Research in Surgical Outcomes and Effectiveness, University Hospital Cleveland Medical Center, Cleveland, OH, USA; Department of Colorectal Surgery, The Royal Wolverhampton NHS Trust, Wolverhampton, UK; Association of Coloproctology of Great Britain & Ireland Patient Liaison Group, UK; Department of Colorectal Surgery, University of California Davis, Sacramento, CA, USA; Department of Medical Education, University of Aberdeen, Aberdeen, UK; Department of Colorectal Surgery, Raigmore Hospital, Inverness, UK; Department of Medical Education, University of Aberdeen, Aberdeen, UK; Medical Education Research and Scholarship Unit, Lee Kong Chian School of Medicine, Nanyang Technological University, Singapore; Department of General Surgery, Royal Alexandra Hospital, Paisley, UK; School of Medicine, Dentistry and Nursing, University of Glasgow, Glasgow, UK

## Abstract

**Background:**

Decision-making under uncertainty may be influenced by an individual’s personality. The primary aim was to explore associations between surgeon personality traits and colorectal anastomotic decision-making.

**Methods:**

Colorectal surgeons worldwide participated in a two-part online survey. Part 1 evaluated surgeon characteristics using the Big Five Inventory to measure personality (five domains: agreeableness; conscientiousness; extraversion; emotional stability; openness) in response to scenarios presented in Part 2 involving anastomotic decisions (i.e. rejoining the bowel with/without temporary stomas, or permanent diversion with end colostomy). Anastomotic decisions were compared using repeated-measure ANOVA. Mean scores of traits domains were compared with normative data using two-tailed *t* tests.

**Results:**

In total, 186 surgeons participated, with 127 surgeons completing both parts of the survey (68.3 per cent). One hundred and thirty-one surgeons were male (70.4 per cent) and 144 were based in Europe (77.4 per cent). Forty-one per cent (77 surgeons) had begun independent practice within the last 5 years. Surgeon personality differed from the general population, with statistically significantly higher levels of emotional stability (3.25 *versus* 2.97 respectively), lower levels of agreeableness (3.03 *versus* 3.74), extraversion (2.81 *versus* 3.38) and openness (3.19 *versus* 3.67), and similar levels of conscientiousness (3.42 *versus* 3.40 (all *P* <0.001)). Female surgeons had significantly lower levels of openness (*P* <0.001) than males (3.06 *versus* 3.25). Personality was associated with anastomotic decision-making in specific scenarios.

**Conclusion:**

Colorectal surgeons have different personality traits from the general population. Certain traits seem to be associated with anastomotic decision-making but only in specific scenarios. Further exploration of the association of personality, risk-taking, and decision-making in surgery is necessary.

## Teaser

This international study has demonstrated that the surgeon’s personality is an independent factor influencing variation in surgical practice. Our findings validate previous work demonstrating that colorectal surgeons possess a different personality trait profile than the general population, with surgeons possessing traits that patients have previously identified as preferable (emotional stability and conscientiousness).

## Introduction

Personality is defined as ‘the dynamic and organized set of characteristics possessed by a person that uniquely influences their cognitions, motivations and behaviours in various situations’^1^. Personality may be objectively measured using validated self-reported measures^2^. While personality is largely stable throughout the lifespan, it is possible to modify aspects of one’s personality, following exposure to experience and/or through changes in beliefs and values^3,4^. The relationship between personality and decision-making is well established in non-medical vocations involving risk and uncertainty, and is strongly predictive of work behaviours across cultures^5^. Across multiple industries, including astronautics^6^, the military^7,8^, and business^9^, personality has been found to influence decision-making. Within the medical profession, personality testing has largely focused on selection and attrition rates from undergraduate or postgraduate medical programmes, and the prediction of examination outcomes^10–12^. However, there has been limited work within medicine exploring how personality may influence clinical decision-making in scenarios that lack a gold standard^13–15^.

Anastomotic decision-making in rectal cancer is complex and is an important example of how decision-making can affect patient care. The three choices to consider are the formation of a primary anastomosis alone, a primary anastomosis with a temporary loop ileostomy (including the later decision to close the stoma), and permanent end colostomy following diversion of the bowel (i.e. no anastomosis). Each decision has specific short- and longer term implications for both the clinician and patient that may impact quality of life, bowel function, and surgery-specific complications^16–21^. Established patient factors in anastomotic decision-making, such as significant comorbidity and frailty^22–24^, do not wholly account for the substantial variation in surgical practice reported from national databases such as the National Bowel Cancer Audit (NBOCA)^25^. Previous work has suggested that surgeon personality^14^, surgeons with a self-belief of possessing lower anastomotic leak rates, and older surgeons, are more likely to form primary anastomosis alone (the higher-risk option) and form fewer stomas^26,27^. Further exploration in a larger sample across cultures is warranted to determine how surgeon personality influences the anastomotic decision and explore the surgeon’s risk perception of anastomotic leakage.

The primary aim of this work was to determine if a relationship existed between surgeon personality and rectal anastomotic decision-making; the secondary aim was to assess if certain trains are ‘beneficial’ for patient care and postoperative outcomes. The hypothesis was that surgeon personality may influence anastomotic decision-making, particularly those traits which may influence the individual surgeon’s risk perception of anastomotic leakage.

## Methods

This was a quantitative study using a cross-sectional design and a survey for data collection. Ethical approval was obtained from the University of Aberdeen College Ethics Review Board (CERB/2020/4/1984).

### Participants

Surgeons from any country who met the following criteria were invited to participate: fully certified colorectal surgeons who independently perform more than 10 elective colorectal cancer operations per year and contribute to multidisciplinary team discussions (as per Association of Coloproctology of Great Britain and Ireland (ACPGBI) and National Institute for Health and Care Excellence (NICE) guidelines at time of ethical approval^28,29^. Exclusion criteria included trainees/residents who had not yet completed their training, non-colorectal specialty general surgeons, or surgeons who did not meet the above definition of a colorectal cancer surgeon. Informed consent was obtained prior to survey commencement. Data were stored securely in accordance with the General Data Protection Regulation (EU-2016/789) and the UK Data Protection Act.

### Recruitment and dissemination

Recruitment was performed via social media (including Twitter via @plato_project) and invitational e-mails from professional bodies such as the ACPGBI.

### Data collection

The survey was available in electronic format via Snap11 Professional and reported using the CHERRIES Checklist (*Appendices S1*)^30^. Following a pilot study on four general surgery trainees to check for readability and repetition, the survey was divided into two parts (defined below) to reduce the time taken for each part (facilitating participation) and allowed participants undertaking Part 2 to complete the scenarios ‘fresh’, to mitigate the influence of social desirability response bias. Part 1 was open for a period of 12 weeks (14 August 2020 to 5 November 2020), subsequently closed to new participants; Part 2 was then opened for 12 weeks (6 November 2020 to 29 January 2021). Only participants who completed Part 1 could participate in Part 2. Reminders were sent at regular intervals via social media and e-mail invites from those registered for participation. Participation was incentivized by e-mailing individual results from the personality survey to those who had completed both parts. As participants submitted identifying information, duplicate entries were identified and removed. Survey participants could only scroll forwards and back, without a ‘review’ page prior to survey completion.

#### Part 1

Part 1 contained data items on demographics (age range, sex, years of experience, country of practice) and the 44-item validated personality tool based upon the Five Factor Model of personality—the Big Five Inventory (BFI; *Appendix S2*)^31,32^. These demographics were of specific interest given evidence from the general population that personality traits change with age^33,34^ and women tend to have higher levels of agreeableness and lower levels of emotional stability than men, findings which persist across cultures^35,36^. In surgeon populations, increasing experience has been associated with increased risk-taking^26,27^.

The BFI has a large evidence base and high level of validity^32^, encompassing personality into five broad domains: openness to experience (degree of originality, creativity); conscientiousness (degree of diligence, planful, rule-following); extraversion (sociability, assertiveness); agreeableness (degree of ability to get on well with others, conflict avoidance, modesty); and emotional stability (even-temperedness *versus* neuroticism)^2,37^. Each domain is considered a spectrum, with low, average, or high levels of each trait expressed by an individual. The BFI scale scores each domain between 1 (low levels of trait) to 5 (high levels of trait). Some items require reverse scoring. The final domain score is calculated from the mean of standard and reversed scored items. BFI tests which have six or fewer missing answers are still valid, provided they are from a spread of domains^32^.

#### Part 2

Part 2 contained hypothetical clinical scenarios involving anastomotic decision-making (*Appendices S3 and S4*). The scenarios were split into two themes: ‘surgeon factors’, where surgeons ranked each scenario between 1 and 10 (where 1 was extremely unlikely to influence decision-making and 10 was extremely likely (*Appendices S3*)); and ‘patient factors’—10 scenarios with ‘drop-down’ options differing for each scenario based on relevance (*Appendices S4*). All scenarios were ambiguous to explore equipoise. Each hypothetical patient scenario was written based upon the personal experiences of steering group members, which included patient and public involvement representation, and stratified by seven colorectal surgeons into high-, medium-, or low-risk options. Steering group consensus of risk was taken as 70 per cent (i.e. a minimum of five surgeon steering group members in agreement), in accordance with previous work^38^. For a limited number of scenario options, the risk-stratification category with the greatest frequency of steering group votes was used as the consensus option, as the small number of steering group members meant it was difficult to achieve consensus in all scenarios. This may reflect the hypothesized variation in practice among experts.

All options for anastomotic decisions were clinically acceptable options to explore equipoise within rectal cancer decision-making. Surgeons who contributed to writing the scenarios were excluded from the final analysis. Examples of scenarios relating to surgeon factors included decision-making following recent personal or witnessed critique after a significant postoperative complication, a recent ‘good run’ of no anastomotic leak, and working with unfamiliar colleagues (*Appendix S3*). Examples of scenarios relating to patient factors included strong patient preferences regarding stomas; impending obstruction or advanced disease at time of presentation; and unexpected intraoperative events such as pelvic bleeding or ureteric injury (*Appendices S3 and S4*).

### Statistical analysis

All effect size estimates for power calculations were derived from a previous pilot study^14^. The mean correlation between personality and decision-making is 0.37; therefore, power calculation determined that a minimum of 52 participants was necessary to achieve a power of 0.80, with an α (two-tailed) of 0.05. Spearman’s rho and comparison across decision-making scenarios were used for repeated-measure ANOVA. One-sample *t* tests were used to compare mean scores of personality traits compared to normative data (*Table 2*)^34,39^. All tests were two-tailed (Pearson’s χ^2^ test) using SPSS Statistics for Windows (Version 27; IBM, Armonk, NY, USA).

## Results

### Demographics

In total, 186 certified colorectal surgeons participated in the personality testing with 127 (68.3 per cent) completing both parts (i.e. also completed the anastomotic scenarios). Altogether, 131 participants were male (70.4 per cent), with 77 surgeons (41.4 per cent) becoming fully qualified within the last 5 years. Surgeons from 22 countries completed Part 1, with the majority practising in Europe (78 in the UK (41.9 per cent); 43 in Western Europe (23.1 per cent); 23 in Eastern Europe (12.4 per cent)) (*Table 1*).

**Table 1:** Demographics of colorectal surgeons in the Plato Project

Demographics	Number of participants (*n* = 186)
**Sex**	
Male	131 (70.4)
Female	54 (29.0)
Prefer not to say	1 (0.5)
**Age (years)**	
30–39	61 (32.8)
40–49	82 (44.1)
50–59	36 (19.4)
60–69	5 (2.7)
70+	2 (1.1)
**Certification experience**	
Within 12 months	21 (11.3)
1–3 years	22 (11.8)
3–5 years	34 (18.3)
5–10 years	48 (25.8)
10–15 years	29 (15.6)
15–20 years	16 (8.6)
20 + years	16 (8.6)
**Region of practice**	
UK	78 (41.9)
Western Europe	43 (23.1)
Eastern Europe	23 (12.4)
North America	10 (5.4)
Australasia	10 (5.4)
Central Asia	9 (4.8)
Rest of the World	13 (7.0)

Data are *n* (%).

### Surgical decisions and personality

No participants were excluded owing to incompletion of BFI items. Surgeons scored higher than worldwide general populations for emotional stability (3.25 *versus* 3.00; *P* <0.001) and had lower levels of agreeableness (3.03 *versus* 3.56; *P* <0.001), extraversion (2.81 *versus* 3.38; *P* <0.001), and openness (3.19 *versus* 3.70; *P* < 0.001)^34,39^ (*Tables 2 and 3*). There were no differences in conscientiousness in comparison to the general population. For context, the worldwide demographics of general population personality traits are summarized in *Table 2*^34,39^.

**Table 2:** Mean surgeon Big Five Inventory Scores *versus* selected world population samples*^,†^

Domain	Colorectal surgeons	Eastern Europe ^39^	Western Europe^39^	USA^34^	East Asia^39^	Middle East^39^	Oceania^39^	One Sample *t* test
**Extraversion**	2.81	3.42	3.38	3.24	3.16	3.40	3.42	*t* _(185)_ = −18.97, *P* <0.001
**Agreeableness**	3.03	3.54	3.56	3.89	3.40	3.78	3.66	*t* _(185)_ = 36.34, *P* <0.001
**Conscientiousness**	3.42	3.38	3.40	3.79	3.08	3.58	3.60	*t* _(185)_ = −7.63, *P* <0.001
**Emotional stability**	3.25	2.92	3.00	3.1	3.30	2.98	2.94	*t* _(185)_ = 12.99, *P* <0.001
**Openness**	3.19	3.72	3.70	3.89	3.34	3.78	3.70	*t* _(185)_ = −20.97, *P* <0.001

*5-point scale—minimum 1 (low levels); maximum 5 (high levels). **^†^**Further regional comparisons are available via Schmitt reference.

**Table 3:** Association between personality and demographics

Variable		Extraversion	Openness	Conscientiousness	Agreeableness	Emotional stability
**Sex**	Female (*n* = 54)	2.75 (0.34)	3.06 (0.34)	3.37 (0.43)	3.03 (0.26)	3.25 (0.32)
	Male (*n* =131)	2.83 (0.29)	3.24 (0.28)	3.44 (0.40)	3.02 (0.27)	3.25 (0.29)
		*P* = 0.066	*P* = 0.001	*P* = 0.278	*P* = 0.770	*P* = 0.961
**Age range (years)**	30–39 (*n* = 61)	2.85 (0.32)	3.27 (0.32)	3.43 (0.36)	2.99 (0.25)	3.26 (0.27)
	40–49 (*n* = 82)	2.82 (0.28)	3.19 (0.31)	3.43 (0.43)	3.02 (0.28)	3.23 (0.32)
	50–59 (*n* = 36)	2.73 (0.35)	3.08 (0.28)	3.31 (0.44)	3.09 (0.26)	3.25 (0.31)
	60+ (*n* = 7)	2.69 (0.26)	3.10 (0.20)	3.71 (0.24)	3.00 (0.30)	3.36 (0.18)
		*P* = 0.215	*P* = 0.019 59-59 < 30.39	*P* = 0.092	*P* = 0.405	*P* = 0 .717
**Years of practice**	Early-career surgeon (<5 years; *n* = 77	2.88 (0.28)	3.23 (0.33)	3.46 (0.35)	2.99 (0.24)	3.28 (0.30)
	Established surgeon (>5 years; *n* = 109)	2.76 (0.32)	3.16 (0.29)	3.39 (0.45)	3.05 (0.28)	3.23 (0.29)
		*P* = 0.006	*P* = 0.063	*P* = 0.152	*P* = 0.093	*P* = 0.141

Specific traits influenced anastomotic decision-making in some settings. Higher rates of stoma formation were associated with higher levels of openness when providing a second opinion (*P* <0.050, Scenario 6—*Appendix S3*). Extraverted surgeons were more likely to have their anastomotic decision-making influenced when operating on a colleague (Scenario 6, *Appendices S3*; ρ = 0.192, *P* = 0.030) (*Table 4*). Variation in practice among experts was confirmed by varied responses to patient-specific scenarios (*Table 5*)—there were only three scenarios where surgeons almost unanimously agreed to stoma formation (Scenarios 2, 4, and 5, *Appendix S4*).

**Table 4:** Correlations with personality and surgeon factor anastomotic scenarios (*Appendix 3*)

		Scenarios	1	2	3	4	5	6	7	8	9	10
**Spearman’s rho**	Extraversion	Correlation Coefficient	0.112	0.134	−0.067	0.043	0.156	.192*	0.132	0.082	0.115	0.106
		Sig. two-tailed	0.206	0.132	0.453	0.628	0.079	0.3	0.137	0.356	0.197	0.231
		Number (*n*)	129	128	128	129	127	128	128	128	128	129
	Male	Correlation Coefficient	.246*	.210*	−0.047	0.1	0.109	*.248	0.159	0.106	0.156	0.062
	Female	Sig. two-tailed	0.018	0.046	0.654	0.343	0.303	0.018	0.132	0.316	0.14	0.555
		Number (*n*)	92	91	92	92	91	91	91	91	91	92
		Correlation Coefficient	−0.068	0.025	−0.107	−0.07	.353*	0.088	0.108	0.134	0.151	0.265
		Sig. two-tailed	0.691	0.881	0.536	0.679	0.035	0.603	0.526	0.43	0.371	0.113
		Number (*n*)	37	37	36	37	36	37	37	37	37	37
	Agreeableness	Correlation Coefficient	0.12	−0.01	−0.41	0.056	.0.10	0.015	0.047	−0.82	0.002	0.05
		Sig. two-tailed	0.895	0.908	0.649	0.526	0.908	0.87	0.602	0.355	0.978	0.577
		Number (*n*)	129	128	128	129	127	128	128	128	128	129
	Male	Correlation Coefficient	−0.064	−0.027	0.017	0.103	0.103	−0.036	0.018	−0.121	−0.047	0.034
	Female	Sig. two-tailed	0.542	0.801	0.876	0.331	0.331	0.734	0.865	0.253	0.655	0.748
		Number (*n*)	92	91	92	92	92	91	91	91	91	92
		Correlation Coefficient	0.165	−0.032	−0.211	−0.039	0.144	−0.032	0.089	0.008	0.111	0.102
		Sig. two-tailed	0.329	0.853	0.217	0.818	0.401	0.851	0.602	0.962	0.512	0.547
		Number (*n*)	37	37	36	37	36	37	37	37	37	37
	Conscientious-ness	Correlation Coefficient	−0.058	−0.076	−0.056	−0.043	−0.082	−0.131	−0.082	−0.039	−0.051	−0.018
		Sig. two-tailed	0.517	0.396	0.531	0.628	0.362	0.141	0.355	0.659	0.568	0.836
		Number (*n*)	129	128	128	129	127	128	128	128	128	129
	Male	Correlation Coefficient	0.018	0.029	−0.075	0.022	−0.089	0	0.028	0.033	−0.008	−0.023
	Female	Sig. two-tailed	0.862	0.783	0.478	0.835	0.4	0.998	0.789	0.758	0.758	0.83
		Number (*n*)	92	91	92	92	91	91	91	91	91	92
		Correlation Coefficient	−0.073	−0.215	0.075	−0.18	−0.034	−.342*	−0.294	−0.121	−0.03	−0.006
		Sig. two-tailed	0.668	0.202	0.664	0.286	0.845	0.038	0.077	0.474	0.86	0.973
		Number (*n*)	37	37	36	37	36	37	37	37	37	37
	Emotional stability	Correlation Coefficient	0.132	−0.025	−0.032	−0.101	0.134	0.077	0.057	0.006	0.081	−0.41
		Sig. two-tailed	0.135	0.78	0.722	0.254	0.134	0.389	0.525	0.95	0.366	0.647
		Number (*n*)	129	128	128	129	127	128	128	128	128	128
	Male	Correlation Coefficient	.207*	−0.017	−0.031	−0.055	0.033	0.099	0.078	−0.03	0.073	0.089
	Female	Sig. two-tailed	0.048	0.87	0.766	0.6	0.755	0.349	0.46	0.78	0.493	0.401
		Number (*n*)	92	91	92	92	91	91	91	91	91	92
		Correlation Coefficient	−0.187	−0.134	−0.059	−0.194	.358*	−0.029	−0.08	0.078	0.006	0.252
		Sig. two-tailed	0.268	0.429	0.734	0.25	0.032	0.865	0.638	0.647	0.973	0.133
		Number (*n*)	37	37	36	37	36	37	37	37	37	37
	Openness	Correlation Coefficient	−0.107	−0.168	−0.051	0.004	0.021	−0.172	−0.016	−.178*	−0.058	−0.041
		Sig. two-tailed	0.228	0.059	0.568	0.966	0.814	0.052	0.858	0.045	0.512	0.647
		Number (*n*)	129	128	128	129	127	128	128	128	128	129
	Male	Correlation Coefficient	0.021	−0.044	−0.092	0.06	0.098	−0.121	0.045	−0.067	−0.022	0.06
	Female	Sig. two-tailed	0.842	0.676	0.382	0.567	0.354	0.255	0.675	0.528	0.835	0.573
		Number (*n*)	92	91	92	92	91	91	91	91	91	92
		Correlation Coefficient	0.013	−0.242	0.202	−0.109	−0.085	−0.311	−0.042	−.408*	0.109	−0.248
		Sig. two-tailed	0.937	0.149	0.236	0.522	0.624	0.061	0.807	0.012	0.52	0.139
		Number (*n*)	37	37	36	37	36	37	37	37	37	37

*Correlation is significant at the 0.05 level (two-tailed).

**Table 5:** Surgeon responses to risk-taking scenarios (*Appendix 4*)

Scenarios	1	2	3	4	5	6	7	8
**Low risk**	94 (73.4)	2 (1.7)	41 (36.0)	4 (3.1)	4 (3.2)	62 (49.6)	39 (37.9)	7 (6.0)
**Medium risk**	33 (25.8)	96 (79.3)	52 (45.6)	125 (96.9)	109 (87.9)	46 (36.8)	60 (58.3)	87 (75.0)
**High risk**	1 (0.8)	23 (19.0)	21 (18.4)	NA	11 (8.9)	17 (13.6)	4 (3.9)	22 (19.0)
**Total**	128	121	114	129	124	125	103	116

Data are *n* (%).

### Surgical decisions and gender

Of the five personality traits, only openness to experience differed between male and female surgeons (131 *versus* 54), with female surgeons having significantly lower levels (3.06 *versus* 3.25; *P* < 0.001). These findings are summarized in *Tables 3 and 4*.

In total, 127 surgeons completed the anastomotic scenarios; 90 were male. Of the scenarios investigating surgeon factors (*Appendix S3*), female surgeons were significantly more likely than males to be influenced by recent personal criticism at a departmental meeting regarding an anastomotic decision where the patient leaked but survived (*P* <0.010) and when they witnessed a colleague being criticized for the same scenario (*P* = 0.020). Male surgeons with higher levels of extraversion were significantly more likely than females to be influenced by criticism at a recent morbidity and mortality meeting following an anastomotic leak (*P* = 0.018) or following a recent unexpected elective mortality from an anastomotic leak (*P* = 0.046).

Only one scenario investigating patient factors and surgeon risk-taking had a significant effect for gender (*Appendix S4*), with male surgeons reporting an increased likelihood of selecting the high-risk option (primary anastomosis; no stoma) for Scenario 2, *Appendix S4* ($\chi_{\left(2\right)}^{2}$ = 10.02, *P* = 0.007), where the patient had a low rectal cancer (close to sphincters) and partial response to neoadjuvant chemoradiotherapy.

### Surgical decisions and age/experience

When comparing surgeon age with personality, the only BFI personality trait difference was found in openness to experience, with surgeons aged 30 to 39 years having higher levels of openness (3.27) than surgeons aged 50 to 59 years who had lower levels (3.08) (*Table 3*). Early career surgeons (qualified within last 5 years) had higher levels of extraversion than surgeons with established practice (2.88 *versus* 2.76; *P* = 0.006).

Surgeons who were highly influenced in their decision-making by a recent ‘good run’ of no anastomotic leaks (Scenario 5, *Appendix S3*), tended to be younger in age (*ρ* = −0.190 *P* = 0.033). In situations where a close colleague had recently been heavily criticized for an anastomotic leak where the patient died (Scenario 7, *Appendix S3*) or when the patient survived (Scenario 9, *Appendix S3*), surgeons with less experience were more likely to be influenced in their anastomotic decision-making based on their colleague’s experiences (*ρ* = −0.260 (*P* = 0.003) and *ρ* = −0.237 (*P* = 0.007), respectively). Surgeons with less experience were highly influenced by recent personal criticism of anastomotic leakage where the patient survived (Scenario 1, *Appendix S3*) and following an unexpected death following an anastomotic leak (Scenario 2, *Appendix S3*) (*ρ* = −0.229 (*P* = 0.009) and *ρ* = −0.214 (*P* = 0.015), respectively).

No correlation was seen between experience (i.e. early career surgeons *versus* established surgeons) with risky choices within the patient factors scenarios (*Appendix S4*). However, increasing age was associated with higher risk-taking in the emergency setting, including where a patient with a mid-rectal cancer presents with impending obstruction and liver metastases (Scenario 3, *Appendix S4*), where surgeons aged 50–59 years were significantly more likely to perform anastomose primarily without stoma formation ($\chi_{\left(6\right)}^{2}$ = 13.04, *P* = 0.041).

## Discussion

This international survey has demonstrated that variation in surgical decision-making is influenced by the personality of the surgeon. Variation in surgical practice was confirmed by consensus about anastomotic decision-making in only three scenarios (scenarios 2, 4, and 5—*Appendix S4*), where there was unanimous agreement to form a stoma. Two of these scenarios indicated strong patient preferences for stoma avoidance that the surgeons overruled. However, this is not to suggest that surgeons do not consider the patient’s wishes important—rather they considered the documented risk of poorer bowel function or anastomotic leak risk to be of greater importance than the risk of forming a stoma. All personality traits may be beneficial and/or detrimental when subjected to specific settings or environmental circumstances (termed trait activation theory)^11^, which may explain our finding that there was no single unifying personality trait which influenced primary anastomosis, temporary stoma formation, or permanent diversion with colostomy across all scenarios, but that specific traits influenced individual scenarios (e.g. openness influenced decision-making when providing a second opinion). Interestingly, patients have previously indicated that they believe the surgeon’s personality influences their perioperative care, identifying high levels of emotional stability and conscientiousness as preferable^40^. While this study demonstrated that surgeons appear to possess these traits, a direct relationship between these specific traits and postoperative outcomes was not established.

Colorectal surgeons had higher levels of emotional stability (even-temperedness) than the general population and possessed lower-than-average levels of agreeableness (tendency towards conflict), extraversion (tendency towards enthusiasm, assertiveness), and openness to experience (tendency towards fixed thinking, routine), with some support for our findings from a recent systematic review on abdominal surgeon personality (high levels of conscientiousness)^15^. Interestingly, female surgeons had lower levels of openness than male surgeons, differing from what is commonly found in the general population^35,36^. Thus, this study builds upon previous work demonstrating that colorectal surgeons may have differing personality traits to the general population^14,15^, while demonstrating that the surgeon’s personality is an independent factor influencing variation in decision-making—a novel finding. The finding that early-career surgeons and female surgeons are highly influenced by recent personal or witnessed criticism in anastomotic decision-making highlights the importance of a supportive working environment, particularly in the morbidity and mortality meeting setting^41^. This may be a result of cognitive appraisal (contributing to anecdotal experience), where the personal interpretation of an event influences the emotional response from the individual^42,43^. With increasing experience, surgeons report less intraoperative stress and subsequently improved performance than less-experienced colleagues, which may explain the susceptibility to criticism in early-career surgeons^44^. Given this information, early-career surgeons and female surgeons are perhaps more likely to benefit from appropriate mentorship throughout their career^45^.

This is the first study to report on a global cohort of colorectal surgeons according to gender in relation to personality traits. While a recent study suggested that the surgeon’s gender accounted for variation in patient postoperative outcomes^46^, this work suggests this may be an oversimplification, with differences arising from the individual’s risk perceptions and inherent personality traits as well as surgeon demographics in the face of specific clinical situations. Surgical decision-making (and thus postoperative outcomes) is likely to be far more complex than the gender of those involved. Interestingly, the study by Wallis *et al*.^46^, which suggested that surgeon gender influences patient outcomes failed to demonstrate this in emergent surgery—a setting where cases are allocated irrespective of training, age, experience, and gender. High-risk decisions with uncertainty are likely to be influenced by the subjective perceptions of the surgeon and their comfort of risk-taking. For example, risk-taking is influenced by the characteristics of the person (including personality and demographics), the specific situation, and the perceived reward from taking that risk^47^. While risk-taking and personality are inter-related, they are separate constructs and the relationship between risk-taking and anastomotic decision-making merits further investigation.

Personality has been demonstrated to change throughout one’s medical career: from medical students throughout their undergraduate degree^48^, postgraduate training^49^, and following retirement^50^. Therefore, the personality changes seen throughout one’s life could be hypothesized to be a cumulative result of life experiences, for example increasing clinical experience in response to various ‘successes’ or ‘failures’ in a surgical career^3,50,51^, and may explain the relationship between experience and decision-making in response to an anastomotic leak. Periodic personality testing could therefore increase the awareness among surgeons of the implications of the individual’s personality on clinical risk-taking and potentially influence patient outcomes.

### Limitations

With any opt-in survey, the selection bias of participants may be present. Responses may be subject to social desirability bias or the participant’s lack of insight into their ‘true self’. However, psychometric testing is generally considered to be reliably answered in ‘non-examined’ unpressurized circumstances^52^. As invitations were distributed via social media, the true response rate is incalculable, and social media may have recruited relatively younger surgeons more interested in personality trait analysis. In addition, the imbalance of surgeons participating from all over the world meant that there were relatively low numbers per country, and therefore it was not possible to correlate risk-taking decision-making with country of practice. Finally, a significant proportion of surgeons (31.7 per cent) completed Part 1 but did not complete Part 2 of the survey.

## Conclusion

Colorectal surgeons possess personality traits that patients have previously identified as ‘preferable’ (emotional stability and conscientiousness). Surgeon personality influences anastomotic decision-making in certain settings. As risk perception is unique to the individual when exposed to specific circumstances, further work is necessary to determine other key cognitive factors which influence surgical decision-making under uncertainty. Improved understanding of how personality traits and risk-taking preferences may influence decision-making demands further investigation, due to its suspected influence upon shared decision-making with patients and subsequently, post-operative outcomes.

## Collaborators

F. McDermott (Royal Devon & Exeter Foundation NHS Foundation Trust, Exeter, UK); C. Young (Royal Prince Alfred Hospital, Sydney, Australia); N.S. Fearnhead (Addenbrooke's Hospital, Cambridge, UK); J. Tiernan (St James' University Hospital, Leeds, UK); C. Maxwell-Armstrong (Nottingham University Hospitals NHS Trust, Nottingham, UK); N. Baxter (University of Melbourne, Melbourne, Australia).

## Supplementary Material

znac200_Supplementary_Data
