# Supplementary material for: Exploring variation in surgical practice: does surgeon personality influence anastomotic decision-making?
Source: Br J Surg. 2022 Jul 19;109(11):1156–63. doi: 10.1093/bjs/znac200 (PMC10364753; doi:10.1093/bjs/znac200)
Supplement: znac200_Supplementary_Data [file znac200_supplementary_data.docx]

**Appendix S1: Checklist for Reporting Results of Internet E-Surveys (CHERRIES)**

| **Item Category** | **Checklist Item** | **Page** |
| --- | --- | --- |
| **Design** | Describe survey design | 6 |
| **Ethical approval and informed consent process** | IRB approval | 6 |
|  | Informed Consent | 6 |
|  | Data Protection | 6 |
| **Development and pre-testing** | Development and testing | 7 |
| **Recruitment Process and description of the sample having access to the questionnaire** | Open survey vs closed | 6-7 |
|  | Contact mode | 6 |
|  | Advertising the survey | 6 |
| **Survey Administration** | Web/Email | 6 |
|  | Context | 7 |
|  | Mandatory/Voluntary | 7 |
|  | Incentives | 7 |
|  | Time/Date | 7 |
|  | Randomisation of items/questionnaires | N/A |
|  | Adaptive questioning | 8-9 |
|  | Number of Items | 8-9 |
|  | Number of pages / screens | Not reported |
|  | Completeness check | 8 |
|  | Review Step | 8 |
| **Response Rates** | Unique site visitor | N/A |
|  | View Rate | N/A |
|  | Participation rate | N/A |
|  | Completion Rate | 9 |
| **Preventing multiple entries from the same individual** | Cookies used | N/A |
|  | IP Check | N/A |
|  | Log file analysis | N/A |
|  | Registration | N/A |
| **Analysis** | Handling of incomplete questionnaires | 7-8 |
|  | Questionnaires submitted with an atypical timestamp | N/A |
|  | Statistical correction | 9 |

**APPENDIX S2: BIG FIVE INVENTORY PERSONALITY INDEX**

**Here are a number of characteristics that may or may not apply to you. Please select a number next to each statement to indicate the extent to which you agree or disagree with that statement.**

1 = Disagree Strongly

2 = Disagree a little

3 = Neither agree nor disagree

4 = Agree a little

5 = Agree Strongly

**I see myself as someone who is:**

1. Is talkative 23. Tends to be lazy
2. Tends to find fault with others 24. Is emotionally stable, not easily upset
3. Does a thorough job 25. Is inventive
4. Is depressed, blue 26. Has an assertive personality
5. Is original, comes up with new ideas 27. Can be cold and aloof
6. Is reserved 28. Perseveres until the task is finished
7. Is helpful and unselfish with others 29. Can be moody
8. Can be somewhat careless 30. Values artistic, aesthetic appearances
9. Is relaxed, handles stress well 31. Is sometimes shy, inhibited
10. Is curious about many different things 32. Is considerate and kind to almost everyone
11. Is full of energy 33. Does things efficiently
12. Starts quarrels with others 34. Remains calm in tense situations
13. Is a reliable worker 35. Prefers work that is routine
14. Can be tense 36. Is outgoing, sociable
15. Is ingenious, a deep thinker 37. Is sometimes rude to others
16. Generates a lot of enthusiasm 38. Makes plans and follows through with them
17. Has a forgiving nature 39. Gets nervous easily
18. Tends to be disorganised 40. Likes to reflect, play with ideas
19. Worries a lot 41. Has few artistic interests
20. Has an active imagination 42. Likes to cooperate with others
21. Tends to be quiet 43. Is easily distracted
22. Is generally trusting 44. Is sophisticated in art, music or literature

**Scoring (R denotes reverse scored items):**

**Extraversion**: 1, 6R, 11, 16, 21R, 26, 31R, 36

**Agreeableness**: 2R, 7, 12R, 17, 22, 27R, 32, 37R, 42

**Conscientiousness**: 3, 8R, 13, 18R, 23R, 28, 33, 38, 43R

**Emotional** **Stability**: 4, 9R, 14, 19, 24R, 29, 34R, 39

**Openness**: 5, 10, 15, 20, 25, 30, 35R, 40, 41R, 44

**APPENDIX S3: SURGEON FACTORS DELPHI**

The following short scenarios are intended to focus on your instinctive decision-making, with the focus away from specific patient factors. They intentionally provide limited information for this purpose.

When considering decision-making in 'normal', non-COVID times, to what extent do the following scenarios influence your next anastomotic decision? (i.e. to what extent do the factors in each scenario play on your mind for your *next* case?)

*(1 – Extremely unlikely to influence; 5 - Neutral; 10 – Extremely likely to influence)*

- 1. You were heavily criticised at the last morbidity and mortality meeting for performing an anastomosis in an elective patient who had a leak and survived.
  2. Within the last 30 days, you have had an unexpected elective mortality following the death of a patient after an anastomotic leak.
  3. You have a patient who has frequent admissions with acute kidney injury and electrolyte disturbance due to a high output ileostomy after elective surgery 3 months ago. She is currently an inpatient.
  4. You are working with an anaesthetist who you personally have only worked with once before, and from that list, had an unexpected elective patient death – presumed to be from a sudden cardiac event.
  5. Despite being very busy, you haven’t had an anastomotic leak for 12 months.
  6. Your next patient is a nurse on one of the surgical wards in your hospital, who has specifically approached you to take their case on.
  7. Your colleague was heavily criticised at the last department morbidity and mortality meeting following the death of an elective patient whose anastomosis leaked.
  8. A patient (needing a low anterior resection for rectal cancer) has been transferred to your care for a second opinion after having neoadjuvant chemo-radiotherapy. They are lodging a complaint against the first surgeon, but you do not know the details.
  9. Your colleague was heavily criticised at the last department morbidity and mortality meeting for performing an anastomosis in a comorbid elective patient which leaked. The patient survived and is now out of hospital.
  10. You performed an elective Hartmann’s procedure on a frail and comorbid older patient. Somewhat surprisingly, the patient recovered extremely well from surgery with no immediate post-operative problems. This operation was intended to be the definitive treatment (i.e. you are not planning on Hartmann’s reversal). Your next case is very similar.

**APPENDIX S4: PATIENT FACTORS DELPHI**

We present 7 hypothetical scenarios involving patient factors. The scenarios are designed to be ambiguous with only some details provided, therefore they are open to interpretation.

1. A 70-year-old female presents as an emergency with acute lower abdominal pain, localised peritonism to the left iliac fossa, with blood tests demonstrating a C-Reactive Protein level of 320 (normal <20). She smokes, has hypertension and has a BMI of 30. Her CT scan shows extensive fluid and gas with a retroperitoneal upper rectal perforation, with no gross contamination elsewhere within the peritoneal cavity. This is proximal to a stricturing upper 1/3 rectal tumour. At laparotomy a retroperitoneal pelvic collection containing faeces was encountered with the known retroperitoneal upper rectal perforation visualised.

*Primary anastomosis; no stoma
Primary anastomosis; defunctioning ileostomy
Hartmann’s
Stoma formation; no resection
Other*

1. A 57-year-old man who has no documented medical comorbidities, had a low rectal cancer picked up by bowel screening. He has a BMI of 30 and has completed neoadjuvant chemoradiation with a marginal/partial response and has confirmed residual disease on MRI 2cm above the sphincters (currently staged as T2). He is absolutely against the idea of a stoma.  
   *Primary anastomosis; no stoma
   Primary anastomosis; defunctioning ileostomy
   Hartmann’s
   Other*
2. An otherwise fit 50-year old male is admitted as an emergency with lower abdominal pain, weight loss and PR bleeding. He states he last opened his bowels yesterday. CT of his chest, abdomen and pelvis demonstrates a stricturing mid-rectal cancer which is not currently; but is at high risk of becoming obstructed. His CT suggests he has 2 liver metastases in the same lobe, which would be technically resectable. MRI confirms mid-rectal tumour that is not margin-threatening.
   *Primary anastomosis; no stoma
   Primary anastomosis; defunctioning ileostomy
   Hartmann’s
   Endoscopic stent
   Stoma formation; no resection
   Other*
3. There have been multiple delays to your operating list, with difficulties getting a high dependency unit bed secured for your 60-year old patient, who has a mid-rectal cancer. You therefore start the case much later than anticipated. During surgery, your trainee was mobilising the sigmoid colon under your supervision. You are called into a colleague’s adjacent operating theatre for a second opinion. While you are out of the room, you give permission for your trainee to continue with the case: “just be careful”. You return to your theatre 15 minutes later. However, it becomes clear to you on closer inspection that the ureter has been inadvertently injured. Urology are called and the ureter is primarily repaired, and a stent is placed. No urine is leaking. The remainder of the dissection and surgery is uneventful.
   *Primary anastomosis; no stoma
   Primary anastomosis; defunctioning ileostomy
   Hartmann’s
   Other*
4. A 38-year old male patient attends clinic with his new partner. He has an impalpable ultra-low rectal tumour and had neoadjuvant chemoradiotherapy given with some response (incomplete). He has consented to a defunctioning loop ileostomy, however this was with great reluctance and after much discussion because of his concerns that surgery may affect his new relationship. His TME was challenging due to pelvic side wall bleeding, which was controlled by packing the pelvis, suture ligating the problematic vessels. He was given a blood transfusion intra-operatively. You are happy that the pelvis is now dry, the resection has been completed satisfactorily and the leak test is negative.

*Primary anastomosis; no stoma; no drain*

*Primary anastomosis; no stoma; pelvic drain placed*

*Primary anastomosis; defunctioning ileostomy; no drain*

*Primary anastomosis; defunctioning ileostomy; pelvic drain placed
Hartmann’s
Other*

1. A 63-year old female (fit and well) is referred as an emergency from her family doctor / GP with constipation, bloating and abdominal pain, and on CT scan is found to have a large bowel obstruction secondary to an upper rectal cancer and no metastases. She is still passing flatus. At the time of surgery, her colon is faecally loaded, which is amenable to on-table lavage. However, the tumour looks to be locally advanced and arises posteriorly. You are concerned that this breaches the posterior TME plane.
   *Primary anastomosis; no stoma
   Primary anastomosis; defunctioning ileostomy
   Hartmann’s*

*Endoscopic stent
Stoma formation; no resection*

*Other*

**Although the focus of our study is in non-pandemic circumstances, for the following 2 scenarios, we are focusing on some COVID-specific cases.**

1. An otherwise fit 50-year old male is admitted as an emergency with lower abdominal pain, weight loss, pyrexia and PR bleeding. He states he last opened his bowels yesterday. CT of his chest, abdomen and pelvis demonstrates a stricturing mid-rectal cancer which is not currently; but is at high risk of becoming obstructed. He had a COVID swab sent due to his temperature, which has returned as a positive result. His CT suggests he has 2 liver metastases in the same lobe, which would be technically resectable.
   *Primary anastomosis, no stoma
   Primary anastomosis with defunctioning ileostomy
   Hartmann’s procedure
   Stoma formation with no resection
   Other*
2. A 57-year old man who has no documented medical comorbidities, had a low rectal cancer picked up by bowel screening. He had symptomatic COVID-19 in the last 4 weeks, has recovered fully and has since had 2 negative swabs as part of his pre-assessment. He has been shielding for 2 weeks. He has a BMI of 30 and has completed neoadjuvant chemoradiation with a marginal/partial response and has confirmed residual disease on MRI 2cm above the sphincters (staging is provisionally T2). He is absolutely against the idea of a stoma.  
   *Primary anastomosis, no stoma
   Primary anastomosis with defunctioning ileostomy
   Hartmann’s procedure
   Stoma formation with no resection
   Other*
3. At the peak of the pandemic thus far in your respective region, to what extent did high population levels of COVID-19 influence your anastomotic decision?
   (1 – Extremely unlikely to influence; 10 – Extremely likely to influence)
4. In this last case, *you* are now the patient. For the purposes of this scenario, you have no significant comorbidities that would preclude you from surgery or that would make you consider a Hartmann’s procedure.

You have a mid-rectal cancer with no metastases, and have had neoadjuvant chemoradiotherapy for margin threatening disease. Prior to your clinic consultation you have made up your mind about what surgery you would prefer. You have not yet approached your consultant colleagues; but have the choice of Surgeon 1 who does not routinely form ileostomies (and tends to anastomose primarily) or Surgeon 2 who frequently forms temporary ileostomies when performing rectal cancer surgery.

Who would you prefer to operate on your rectal cancer?
*Surgeon 1 – tends to anastomose primarily (fewer stomas)
Surgeon 2 – tends to defunction regularly*
